# Dual Function of Glucosamine in Gelatin/Hyaluronic Acid Cryogel to Modulate Scaffold Mechanical Properties and to Maintain Chondrogenic Phenotype for Cartilage Tissue Engineering

**DOI:** 10.3390/ijms17111957

**Published:** 2016-11-23

**Authors:** Chih-Hao Chen, Chang-Yi Kuo, Yan-Jie Wang, Jyh-Ping Chen

**Affiliations:** 1 Department of Chemical and Materials Engineering, Chang Gung University, Kwei-San, Taoyuan 33302, Taiwan; chchen5027@gmail.com (C.-H.C.); onesky1997@hotmail.com (C.-Y.K.); jack96383@hotmail.com (Y.-J.W.); 2 Department of Plastic and Reconstructive Surgery and Craniofacial Research Center, Chang Gung Memorial Hospital, Kwei-San, Taoyuan 33305, Taiwan; 3 Graduate Institute of Health Industry and Technology, Research Center for Industry of Human Ecology, Chang Gung University of Science and Technology, Kwei-San, Taoyuan 33302, Taiwan; 4 Department of Materials Engineering, Ming Chi University of Technology, Tai-Shan, New Taipei City 24301, Taiwan

**Keywords:** glucosamine, hyaluronic acid, gelatin, cross-linking, cryogel, chondrocytes, cartilage tissue engineering, mechanical properties

## Abstract

Glucosamine (GlcN) fulfills many of the requirements as an ideal component in scaffolds used in cartilage tissue engineering. The incorporation of GlcN in a gelatin/hyaluronic acid (GH) cryogel scaffold could provide biological cues in maintaining the phenotype of chondrocytes. Nonetheless, substituting gelatin with GlcN may also decrease the crosslinking density and modulate the mechanical properties of the cryogel scaffold, which may be beneficial as physical cues for chondrocytes in the scaffold. Thus, we prepared cryogel scaffolds containing 9% GlcN (GH-GlcN9) and 16% GlcN (GH-GlcN16) by carbodiimide-mediated crosslinking reactions at −16 °C. The crosslinking density and the mechanical properties of the cryogel matrix could be tuned by adjusting the content of GlcN used during cryogel preparation. In general, incorporation of GlcN did not influence scaffold pore size and ultimate compressive strain but increased porosity. The GH-GlcN16 cryogel showed the highest swelling ratio and degradation rate in hyaluronidase and collagenase solutions. On the contrary, the Young’s modulus, storage modulus, ultimate compressive stress, energy dissipation level, and rate of stress relaxation decreased by increasing the GlcN content in the cryogel. The release of GlcN from the scaffolds in the culture medium of chondrocytes could be sustained for 21 days for GH-GlcN16 in contrast to only 7 days for GH-GlcN9. In vitro cell culture experiments using rabbit articular chondrocytes revealed that GlcN incorporation affected cell proliferation, morphology, and maintenance of chondrogenic phenotype. Overall, GH-GlcN16 showed the best performance in maintaining chondrogenic phenotype with reduced cell proliferation rate but enhanced glycosaminoglycans (GAGs) and type II collagen (COL II) secretion. Quantitative real-time polymerase chain reaction also showed time-dependent up-regulation of cartilage-specific marker genes (COL II, aggrecan and Sox9) for GH-GlcN16. Implantation of chondrocytes/GH-GlcN16 constructs into full-thickness articular cartilage defects of rabbits could regenerate neocartilage with positive staining for GAGs and COL II. The GH-GlcN16 cryogel will be suitable as a scaffold for the treatment of articular cartilage defects.

## 1. Introduction

Articular cartilage has a low regeneration rate owing to lack of vasculature and low cellularity. Therefore, damage to articular cartilage from trauma or disorders usually requires surgical intervention. Nonetheless, conventional repair strategies for articular cartilage are prone to induce the formation of fibrocartilage tissue, which possesses inferior properties. The cartilage tissue engineering approach could serve to produce neocartilage to replace or repair the damaged cartilage at the impaired joint and restore its full function. Such an approach combines cells, scaffolds, and active biomolecules for tissue regeneration. As scaffold materials, natural polymers such as collagen and hyaluronic acid (HA) are often employed to mimic the extracellular matrix (ECM) component of native cartilage. As for the cell source, articular chondrocytes are often used since they are the native, differentiated cell type of articular cartilage. However, culture and expansion of chondrocytes in vitro often leads to changes in chondrocyte phenotype. To promote the re-differentiation of expanded chondrocytes, growth factors such as transforming growth factor-β and insulin-like growth factor are added to the medium, and three-dimensional (3D) culture systems are used [1]. Nonetheless, even with 3D culture systems or growth factor supplementation, the cartilage tissue engineering approach still faces a challenge to maintain the chondrogenic phenotype during chondrocyte culture. 

Traditionally, the preparation of 3D sponge scaffolds based on water-soluble and biodegradable polymers involves freezing a polymer solution by forming ice crystals within the gelled solution, followed by crosslinking the lyophilized matrix with a chemical crosslinker. However, scaffolds prepared through this approach exhibit several drawbacks. Because the concentration of the chemical crosslinking agent exhibits a gradient distribution from the external to the internal portion of the scaffold, both internal and external pores will vary in size after crosslinking [2]. As the external part of the scaffold is exposed to more crosslinking agent, the degree of crosslinking in this area is higher, which leads to smaller pores in the external part of the scaffold. In contrast, larger pores are produced in the internal part of the scaffold. The inconsistent pore size may affect cell migration. If the external pores are too small, cells cannot migrate to the interior, thus limiting the space for cell growth within the scaffold. Furthermore, with the lower degree of crosslinking in the internal part of the scaffold, the internal structure is prone to damage under stress and the ability of the scaffold to withstand stress is reduced.

However, the fabrication process of cryogel could compensate for the aforementioned disadvantages. In contrast to the lyophilization approach, the fabrication of cryogel scaffolds involves adding a crosslinking agent to the solution before freezing. Hence, crosslinking reactions occur while ice crystals form within the scaffold [3]. During cryogel synthesis, the polymer and the crosslinking agent must be mixed evenly to avoid variance in the concentration in the interior and exterior of the scaffold. When the polymer solution reacts at sub-zero temperatures, most of the solution freezes into ice crystals, thereby concentrating the reactants. The crosslinking reaction then proceeds under maximum concentration, forming dense network structures that enhance the mechanical strength of the cryogel [4,5]. The size and distribution of the pores within the cryogel depend on the ice crystals. Because the crosslinking agent and the polymer are completely mixed, pores distribute evenly, and cells can easily migrate inward. Furthermore, when the material is subjected to stress, the force distributes evenly, enabling the scaffold to withstand increased stress. With a reduced crosslinking reaction rate as the temperature falls, the reaction time for cryogel fabrication under sub-zero temperatures must be extended to allow for complete reaction. The fabricated cryogel scaffold has favorable characteristics such as pore interconnectivity, highly porous structure, mechanical stability, and elasticity to be applied as an ideal scaffold for tissue engineering [6,7,8,9].

Gelatin is obtained from collagen in animal skin and tissues such as articular cartilage. The collagen obtained undergoes heat treatment that destroys its helical 3D structure, which further undergoes hydrolysis to produce the water-soluble multipeptide polymer gelatin. Gelatin comprises repeating amino acid sequences, which mainly consist of glycine, proline, and hydroxyproline. Previous studies indicated that the structure of gelatin has the arginine-glycine-aspartic acid (RGD) sequence that allows cells to attach and grow while maintaining their bioactivity [10,11]. Hyaluronic acid is composed of *N*-acetylglucosamine and d-glucuronic acid, which are linked via β-(1,3) bonds through dehydration polymerization. It is a major component of the ECM in connective tissue such as skin and eye lens and is used clinically to treat osteoarthritis [12]. As the HA polymer chain carries substantial negative charges, the molecules of HA completely unfold in water due to charge repulsion, occupying considerable space and enabling HA to absorb water 1000 times its own weight [13]. HA serves as the major constituent of the synovial fluid in the knee joint, whose functions include protecting and lubricating chondrocytes, regulating protein expression in chondrocytes, transporting molecules such as nutrient substrates and metabolites, and stabilizing the network structure of the collagen. In addition, when combined with chondrocytes, HA enables chondrocytes to proliferate and sustain their active form [14]. In normal physiological conditions, HA can activate the CD44 receptor on the chondrocyte surface and modify the signal transduction pathway, thus preventing chondrocytes from de-differentiating [15].

Combining the advantages offered by gelatin and HA towards chondrocytes, gelatin/HA scaffolds (including cryogel scaffolds) have been shown to be suitable for applications in cartilage tissue engineering [16,17,18,19]. Nonetheless, from a tissue engineering point of view, the incorporation of a bioactive signal molecule into gelatin/HA scaffolds to promote chondrogenesis seems to be a rational approach to further enhance their functions. Glucosamine (GlcN) is a simple amino sugar derived from the substitution of a hydroxyl group of a glucose molecule with an amino group. It is synthesized in all organisms, including bacteria, yeast, filamentous fungi, plants, and animals, and mainly produced by acid hydrolysis of chitin extracted from crab and shrimp shells. GlcN can be decomposed into amino acids inside the body and then converted to chondroitin, which is a vital nutrient for chondrocytes. It also stimulates chondrocytes to synthesize more glycosaminoglycans (GAGs) and proteoglycan [20], thus enabling joint cartilage to reach metabolic balance and protecting it from damage. In addition, GlcN was reported to have anti-inflammatory effects [21,22,23]. If hyaline cartilage stores more GlcN, more proteoglycan and collagen can be synthesized. Therefore, more lubricant can be absorbed to maintain the function of the joint. As GlcN treatment enhanced human stem cells chondrogenesis and maintained cartilage matrix gene expression in chondrocytes, it was reported to have chondroprotective properties on cartilage [24]. The treatment of temporomandibular joint disorders and rheumatoid arthritis was attempted using compounds containing GlcN. Pharmacological and biological applications of GlcN have been recently reported for the treatment of cancer, skin disorders, bacterial infection, and cardiovascular diseases [25].

The composition and mechanical properties of cryogel scaffolds could influence chondrocyte behavior [26]. In this paper, we aimed to investigate the dual function of GlcN when it is incorporated into gelatin/HA (GH) cryogel scaffolds, which can affect cell functions and induce tissue regeneration through biological and physical cues. We hypothesized that the incorporation of GlcN into GH cryogel scaffold would influence the crosslinking density of the matrix thus modulating the mechanical properties of the scaffold, which may provide beneficial physical cues for chondrocytes. In addition, the bioactive GlcN released from the cryogel matrix may act as biological cue to maintain the chondrogenic phenotype. We prepared GH-GlcN9 (9 wt % GlcN) and GH-GlcN16 (16 wt % GlcN) cryogels through carbodiimide-mediated crosslinking reactions by cryogelation at a sub-zero temperature. We studied the effects of GlcN on cryogel properties, followed by studies of cell proliferation, morphology, and maintenance of chondrogenic phenotype of chondrocytes in cryogel scaffolds in vitro, and tested the best cryogel scaffold for cartilage regeneration in a rabbit knee joint surface repair model.

## 2. Results and Discussion

### 2.1. Characterization of Cryogels

**Figure 1:**
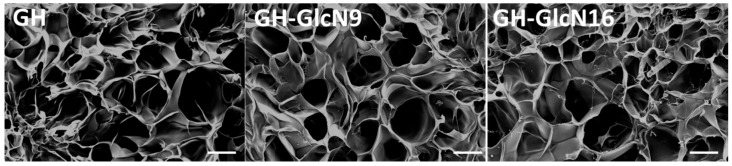
Scanning electron micrographs of GH, GH-GlcN9 and GH-GlcN16 cryogel scaffolds. GH: gelatin/hyaluronic acid cryogel; GH-GlcN9: gelatin/hyaluronic acid/glucosamine cryogel with 9% glucosamine; GH-GlcN16: gelatin/hyaluronic acid/glucosamine cryogel with 16% glucosamine. Scale bar = 100 μm.

Using EDC as the crosslinking agent, the carboxyl group of HA was initially activated in the first step to form an active *O*-acylisourea intermediate that was displaced by nucleophilic attack from the primary amino group of gelatin or GlcN. Since amino groups from both GlcN and gelatin competed for activated carboxyl groups of HA and GlcN contained only one amino group, the addition of GlcN is expected to interrupt the crosslinking reaction between HA and gelatin. Therefore, with the same preparation condition as GH cryogel, we could only fabricate gelatin/HA/GlcN cryogel containing up to 16% GlcN (i.e., GH-GlcN16). The crosslinking reaction was performed at −16 °C to produce ice crystals, which yield pores once they melt. From scanning electron microscopy (SEM) observations, all cryogels exhibit similar interconnected open pore morphology (Figure 1). The interconnectivity of the macro-pores of the scaffold for cartilage regeneration is essential to assure cell seeding or cell invasion from subchondral bone [27]. As pore size is determined by the ice crystal size which is influenced by the crosslinking temperature, we expect the addition of GlcN will not affect the pore size of the scaffold. The average pore size determined from capillary flow porometry is around 100 μm and there is no significant difference in pore size (Table 1). The pore size is also within the range for chondrocyte growth in a 3D scaffold [27]. All synthesized cryogel scaffolds show high porosity, greater than 80%, which is beneficial for cell ingrowth and survival (Table 1). However, incorporation of GlcN results in higher porosity, which depends on GlcN concentration in the order of GH-GlcN16 > GH-GlcN9 > GH. Higher porosity was shown to be beneficial for cell attachment and migration [28]. The density of cryogel shows an inverse relationship with the porosity as expected (Table 1).

The swelling properties of the scaffolds were reported to significantly affect cell behavior, such as adhesion, growth, and differentiation [29]. As shown in Table 1, the incorporation of GlcN drastically influences the swelling of cryogel in water as both GH-GlcN9 and GH-GlcN16 have significantly higher swelling ratio than GH cryogel, which also depends on GlcN concentration. The difference in swelling ratio could not be explained satisfactorily from the cryogel composition as the highly water-absorbing HA in the cryogel varied from 4 to 5 wt %. The level of crosslinking between macromolecules within a 3D scaffold may affect the swelling property of the polymeric scaffold with more crosslinking leading to a less swelling ratio [30]. By substituting gelatin in the cryogel with GlcN, the crosslinking reactions between HA and gelatin will be hindered with single-point attachment of GlcN with HA, which produces a cryogel matrix with a higher swelling ratio. Indeed, GlcN is expected to interrupt the crosslinking network and decrease the crosslinking density within the hydrogel matrix formed between HA and gelatin. This low crosslinking density could unrestrain the swelling of the hydrogels and result in higher swelling ratios.

### 2.2. Cryogel Degradation and GlcN Release

**Figure 2:**
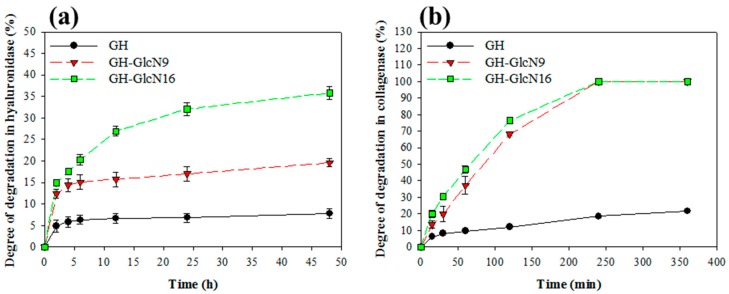
Degradation of GH, GH-GlcN9 and GH-GlcN16 cryogels in (**a**) hyaluronidase and (**b**) collagenase.

The hyaluronidase is an enzyme that leads to HA degradation by randomly cleaving β-*N*-acetylhexosamine-(1,4) glycosidic bonds in HA. The trend of cryogel degradation in hyaluronidase indicated GlcN incorporation increased the rate and degree of degradation in the order of GH-GlcN16 > GH-GlcN9 > GH (Figure 2a). GH-GlcN16 showed ~35% degree of degradation in 2 days. Collagenase is a protease that cleaves the bond between a neutral amino acid (X) and glycine in the sequence Pro-X-Gly-Pro, which is found with high frequency in collagen and gelatin. The degradation of the cryogel in collagenase followed a similar trend as was observed in hyaluronidase with a positive influence from GlcN (Figure 2b). However, a noted difference was the rate of degradation in collagenase. Although the degradation behavior of GlcN9 and GlcN16 was comparable in collagenase, the degree of degradation could reach 100% within 4 h. We inferred that because GlcN is a one-molecule substance consisting of glucose and a nitrogen amine, it could not facilitate crosslinking with HA in the same way as gelatin, which contains multiple primary amino groups from lysine residues. The reduced crosslinking density in the presence of GlcN invariably leads to higher degrees of degradation when the main chains in the crosslinked network are cleaved in the presence of hyaluronidase or collagenase. This effect is more pronounced when using collagenase since gelatin is the major component in the cryogel with >80 wt %.

**Figure 3:**
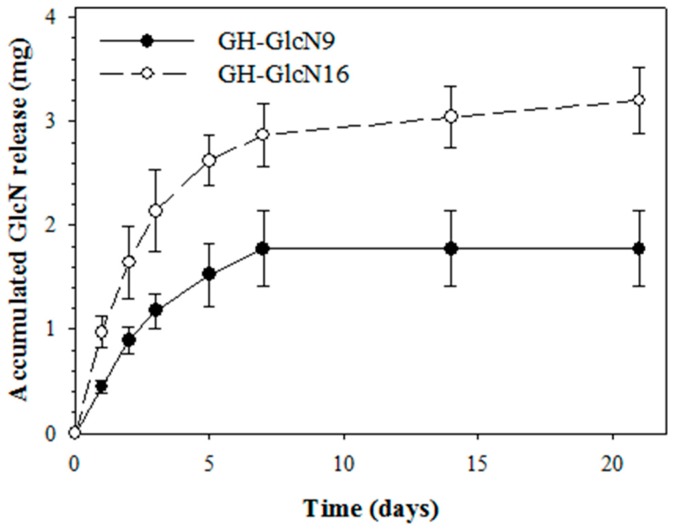
Glucosamine (GlcN) release from GH, GH-GlcN9 and GH-GlcN16 cryogels.

For GlcN release, we used the spent culture medium of chondrocytes at day 3 since chondrocytes proliferation will produce metabolites and enzymes that could lead to cryogel degradation and GlcN release. Therefore, this release experiment will more closely reveal the amount of GlcN that chondrocytes are exposed to during in vitro culture. From the release curves of GlcN, we found that, at the same time points, GH-GlcN16 released more GlcN than GH-GlcN9 (Figure 3). After day 7, the amount of GlcN released by GH-GlcN9 could not be detected in the medium, while GH-GlcN16 continued to release a substantial amount of GlcN. This sustained release of GlcN is important to provide biological cues for maintaining the chondrogenic phenotype in vitro and in vivo. The accumulated GlcN release from GlcN9 and GlcN16 at the end of 21 days was 1.6 and 3.1 mg, respectively, which were 94% and 97% of the amount of GlcN used to prepare the cryogels.

### 2.3. Mechanical Properties of Cryogels

#### 2.3.1. Compression Mechanical Tests

**Figure 4:**
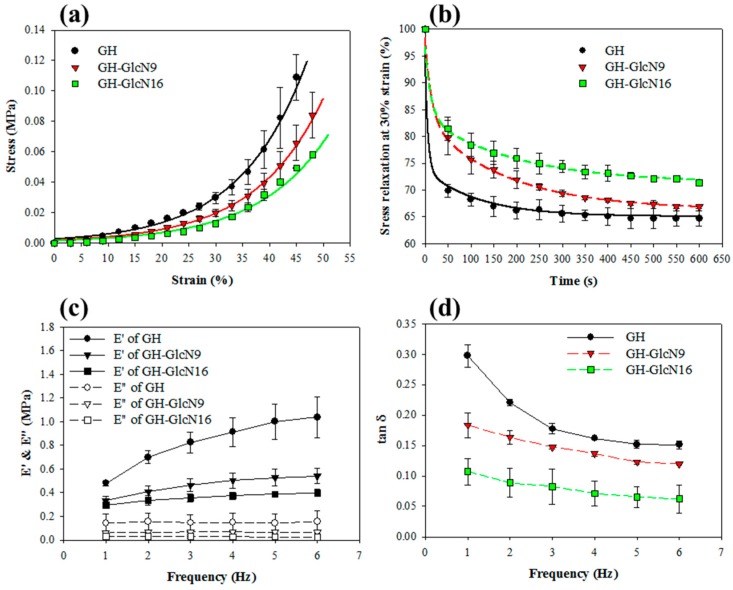
The compressive mechanical properties of GH, GH-GlcN9 and GH-GlcN16 cryogels. (**a**) Stress-strain curve; (**b**) stress relaxation at 30% strain. The viscoelastic properties of cryogels determined by dynamic compression testing at 30% strain are shown as storage modulus (*E*′) and loss modulus (*E*″) (**c**); and loss tangent (tan δ) (**d**).

As shown in Figure 4a, all cryogels showed typical non-linear compressive stress (σ)-strain (ε) curves of covalently crosslinked gels, which are fitted satisfactorily with the empirical exponential equation (Equation (3)). The elastic moduli, calculated from the slopes of the stress-strain curves, changed continuously with strain during compression. The elastic moduli at 10% and 30% deformation (ε = 0.1 and 0.3) were calculated from the fitting equation and listed in Table 2 together with failure strain, failure stress, and toughness. The elastic modulus for GH was 1.5 and 1.8 times that of GH-GlcN9 and GH-GlcN16, respectively, at 10% strain. At 30% strain, the elastic modulus for GH was respectively 1.6 and 1.9 times that of GH-GlcN9 and GH-GlcN16. Overall, the incorporation of GlcN in the cryogel significantly decreased the elastic modulus (stiffness), failure stress and toughness (strain energy to failure) (Table 2). The higher stiffness coincides with the slower degradation rate (Figure 2) from the higher crosslinking degree in GH cryogels. Comparing GH-GlcN9 and GH-GlcN16, only failure stress shows significant difference. However, no statistically significant difference in failure strain among the three cryogels was observed, suggesting that all cryogels could withstand similar degrees of deformation. Therefore, GH was capable of withstanding a greater level of force compared with other groups. The quasi-static compression tests revealed that the mechanical properties of GH were significantly better than those of GH-GlcN9 and GH-GlcN16. With comparable pore size, this effect could be attributed to the extent of the crosslinked network in the cryogel strut.

As shown in previous studies, hydrogels with lower stiffness could maintain better chondrogenic phenotype as chondrocytes in softer hydrogels secreted more collagen and GAGs than in stiffer hydrogels [31,32]. A novel hydrogel possessing a continuous gradient of Young’s modulus was used for chondrocyte culture. Chondrocytes in the hydrogel region with a lower Young’s modulus was found to secrete more ECM than those in the region with a higher Young’s modulus [33]. When chondrocytes were grown in matrices with a Young’s modulus ranging from 4 to 100 kPa, the actin organization and cellular proliferation was the lowest and chondrocytes showed differentiated phenotype in 4 kPa matrices, judging from higher production of type II collagen (COL II) and aggrecan, and lower production of type I collagen [34].

#### 2.3.2. Stress Relaxation Tests

Compressive stress relaxation experiments were used to assess the mechanical stability of cryogels and ensure that cryogels will not deform to an unacceptable level during prolonged static loading (Figure 4b). The percent stress relaxation from 0 to 50 s decreased by 30.2% for GH; by 20.2% for GH-GlcN9; and by 18.6% for GH-GlcN16. The percent relaxation decrease continued until 600 s and the relative remaining stress of GH-GlcN16 is 71.4%, which is significantly higher than that of GH-GlcN9 (66.9%) and GH (64.7%), indicating that incorporation of GlcN could raise the support force and provide more stable mechanical responses for the cryogel when stressed. When a gel is subject to a constant strain, the stress in the gel relaxes by different mechanisms, depending on the types of crosslinks. For a gel with covalent crosslinks, the stress relaxes as water migrates out of the gel, so that the network undergoes elastic deformation. The time scale of the relaxation increases with the size of the sample for the gels with covalent crosslinks. For the gel with covalent crosslinks, the stress relaxed slower when the radius of the disk was larger [35]. The fact that the swelling ratio is in the order of GH-GlcN16 > GH-GlcN9 > GH coincides with this finding as wetted cryogels were used for stress relaxation experiments. Hydrogels developed to repair articular cartilage, for example, may be more effective if their stress relaxation behavior matches that of the native tissue, since such behavior affects transfer of loads and transport of nutrients. It has been reported that cell spreading, proliferation, and osteogenic differentiation of mesenchymal stem cells are all enhanced when cells were cultured in gels with faster relaxation [36]. However, the rapid stress relaxation characters of hydrogels promoted the de-differentiation of chondrocytes as they down-regulated the gene expression of COL II and aggrecan [37].

#### 2.3.3. Dynamic Mechanical Analysis

As cryogel is a viscoelastic solid material, it exhibits both viscous and elastic characteristics when undergoing deformation. Figure 4c exhibits the viscoelastic behavior of cryogel with the storage (*E*′) and viscous (*E*′′) moduli measured using a frequency scan from 1 to 6 Hz. The values of both viscoelastic parameters were significantly higher for GH. In particular, the value of the storage modulus (*E*′) at 6 Hz for GH was 1.04 MPa, whereas the corresponding values for GH-GlcN9 and GH-GlcN16 were 0.54 MPa and 0.40 MPa, respectively. The storage modulus represents the elastic component of a material and is an indicator of the capability of a material to store energy during deformation [38]. The results revealed that GH was capable of storing more energy compared with GH-GlcN9 and GH-GlcN16. A previous study indicated hydrogel with a lower storage modulus could maintain the chondrogenic phenotype better [33].

Figure 4d shows that the loss tangent (tan δ) varied when scaffolds were subjected to various frequencies. The tan δ value revealed information about the viscoelastic properties of the materials [38]. A smaller tan δ indicated that the material had higher elasticity. For all cryogels, tan δ decreased with increased frequency. However, GlcN had the largest loss tangents followed by GlcN9 and GlcN16, indicating adding GlcN enhanced the elasticity of cryogels. Enhanced cartilaginous matrix accumulation in scaffolds with increased elasticity was reported in the literature [39]. 

#### 2.3.4. Cyclic Compression Analysis

**Figure 5:**
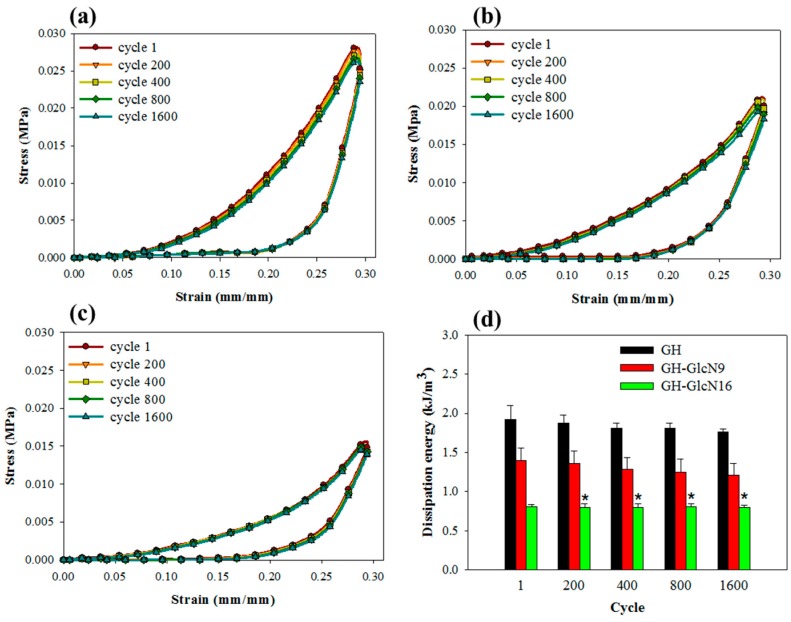
The loading–unloading hysteresis curves during 1600 successive compressions to a maximum strain of 0.3 for (**a**) GH; (**b**) GH-GlcN9; and (**c**) GH-GlcN16 cryogels. (**d**) Dissipation energy during hysteresis of cryogels calculated from the loop area in the loading–unloading hysteresis curve during different compressive cycles. * *p* > 0.05 compared with GH-GlcN16 cycle 1.

We used 1 Hz as the frequency for cyclic compressive testing since the articular cartilage bears loading within the range from 0.1 to 2 Hz [40]. Figure 5a–c reveals a hysteresis loop in the stress-strain curve because the scaffold dissipated energy during the cyclic compression tests. The dissipation energy, represented by the area enclosed within the hysteresis loop, is the amount of mechanical energy dissipated [41]. As shown in Table 2, all cryogels showed energy dissipation percentage similar to that of native cartilage, which ranges from 50% to 80% [42]. The dissipation energy and the percentage of energy dissipation of GH cryogel are higher than those of GH-GlcN9 and GH-GlcN16. Although all cryogels were found to be resilient from dissipation analysis, GH-GlcN16 returned the most energy (40.3%) used to deform them, followed by GH-GlcN9 (38.7%) and GH cryogel (31.8%). GH-GlcN16 cryogel therefore expended less energy than did GH and GH-GlcN9 during deformation, indicating GlcN incorporation leads to a more elastic scaffold, consistent with the lower tan δ value shown in Figure 4d. This is consistent with a previous finding that under an identical dynamic compression, the dissipation was higher for scaffolds crosslinked with a higher amount of crosslinker, hence with a higher crosslinking density [43]. When dynamical loading is present, scaffolds with higher dissipation levels upregulated the gene expression of chondrogenic markers compared with scaffolds with lower dissipation levels [43]. Therefore, incorporation of GlcN in GH cryogel will have a negative impact on chondrogenic markers regulation considering energy dissipation.

As shown from Figure 5a–c, the stress-strain curves resulting from cyclic compression loading during the first cycle and all subsequent cycles were fully reversible, highly reproducible, and encompassed a similar area. From the dissipation energy calculated during each compression cycle, the dissipative energy of the 1600 cycle was 91%, 86%, and 97% of that of the first cycle for GH, GH-GlcN9, and GH-GlcN16, respectively (Figure 5d). Furthermore, there was no significant difference between the dissipation energies during all cycles only for GH-GlcN16, indicating that GH-GlcN16 cryogel can fully recover from compressive loading–unloading cycles and that 30% strain does not cause permanent bond breakage. This unique feature contributes to the development of the GH-GlcN16 cryogel as a tough scaffold for cartilage tissue engineering, which can recover from large strains and absorb impacts without permanent damage.

### 2.4. In Vitro Experiments

#### 2.4.1. Cell Proliferation

**Figure 6:**
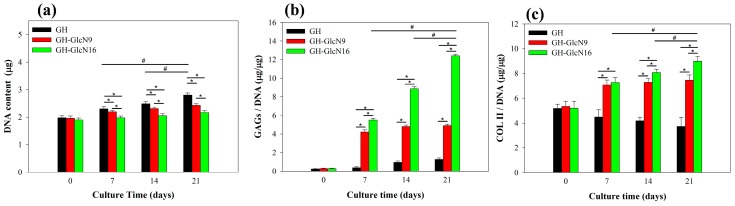
Proliferation and extracellular cellular matrix production of chondrocytes in GH, GH-GlcN9 and GH-GlcN16 cryogels. (**a**) DNA content; (**b**) glycosaminoglycans (GAGs) content normalized to DNA content; (**c**) type II collagen (COL II) content normalized to DNA content. * *p* < 0.05 compared between different cryogels; ^#^ *p* < 0.05 compared between different time points excluding day 0.

The influence of GlcN on cartilage development, focusing particularly on the functions of GlcN in maintaining chondrogenic phenotype, including chondrocyte morphology, gene expression and matrix formation, were determined by means of in vitro cell culture using rabbit chondrocytes [20,21,22,23]. We first examined the attachment and proliferation of chondrocytes using DNA assays. No significant difference in the attached cell number was found from the DNA content at day 0, indicating that GlcN incorporation did not influence cell attachment (Figure 6a). However, the cell proliferation rate showed significant difference between cryogels. Chondrocytes in GH proliferated steadily up to 21 days and the cell number was significantly higher than that in GH-GlcN9 and GH-GlcN16, with the lowest cell number being in GH-GlcN16 (Figure 6a). The reduced cell proliferation is consistent with previous reports that showed growth inhibition of various cells in the presence of GlcN [44,45]. Specifically, prolonged exposure of primary chondrocytes to optimal concentrations of GlcN increased matrix production with concomitant inhibition of chondrocyte proliferation [46]. The GlcN concentration significantly affected cell behavior and adding GlcN led to a concentration-dependent decrease in bovine articular cartilage proliferation in hydrogel [46,47].

#### 2.4.2. GAGs and COL II Production

Proteoglycan is a key component of the cartilage ECM; hence, GAGs content analysis is a key indicator of whether chondrocytes function normally. Quantitatively, GAGs content normalized to DNA content (GAGs/DNA) was the maximum for GH-GlcN16 cryogel, which is significantly different from other groups throughout the culture period (Figure 6b). At day 21, the GAGs production level in GH-GlcN16 was approximately six times as high as that of GH, and that of GH-GlcN9 was approximately three times as high as that of GH. Interestingly, the GAGs production increased with time up to day 21 for GH-GlcN16 while for GH-GlcN9 the GAGs production remained constant after day 7 (Figure 6b). This is in line with the GlcN release profile from the cryogel where GH-GlcN9 could not release any GlcN after day 7 (Figure 3). The stimulating effect of GlcN to promote cartilage to secrete proteoglycan has been reported [48].

In addition to GAGs, COL II is another key indicator of whether chondrocytes function normally. A similar trend in the accumulation of COL II with GAGs was observed (Figure 6c). In particular, the COL II/DNA values of the GH cryogel were significantly lower than other cryogels. Furthermore, the COL II production level of the GH cryogel decreased over time. This phenomenon indicated that chondrocytes de-differentiated in GH cryogel. The COL II production levels of GH-GlcN16 are significantly higher than those of GH-GlcN9 throughout the culture period. Most importantly, the COL II production increased with time only for GH-GlcN16, indicating re-differentiation of chondrocytes in GH-GlcN16. This trend could be also observed in Figure 6b for GAGs/DNA.

Previously, incubation of chondrocyte/scaffold constructs with 2 mM or less GlcN medium resulted in the highest cartilage specific matrix production, GAGs and COL II, but higher amounts of GlcN in the culture medium had adverse effects on chondrocyte matrix production [46]. No stimulatory effect of GlcN on cartilage matrix synthesis was observed at low exogenous GlcN concentrations [49]. Indeed, the released GlcN from the cryogel in the cell culture medium is within the concentration range reported. Thus, by fine tuning the content of GlcN in the cryogel, GlcN released from GH-GlcN16 could exert its stimulating effect toward GAGs and COL II synthesis throughout the culture period.

#### 2.4.3. Live/Dead Staining and SEM Observation

**Figure 7:**
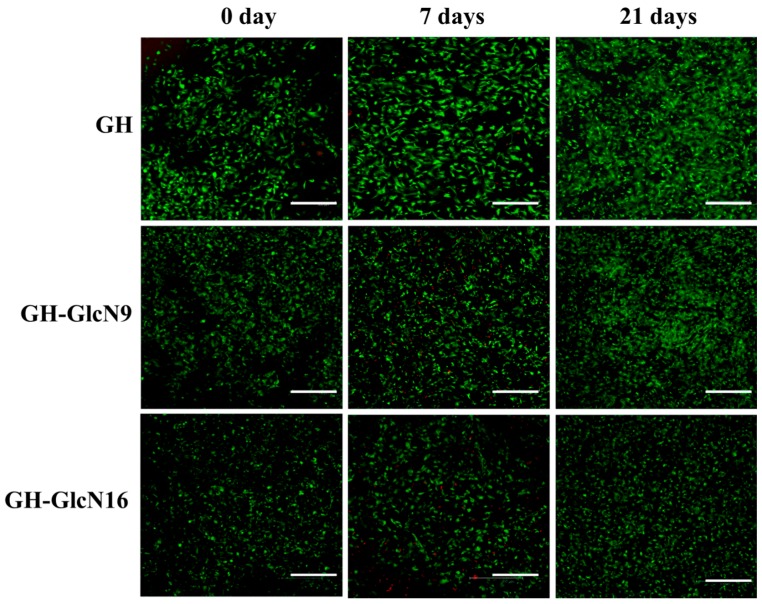
Live/Dead staining of chondrocytes after being cultured in GH, GH-GlcN9 and GH-GlcN16 cryogels for 0, 7, and 21 days. Bar = 300 μm.

The Live/Dead cell viability assay demonstrated high cell viability throughout the 21 day culture period for all cryogels, with most of the chondrocytes being alive and stained green (Figure 7). The number of viable cells increased with time for all groups, indicating GlcN in cryogel did not affect cell viability. However, GH-GlcN16 showed the lowest cell density, which is consistent with the cell proliferation results from DNA assays (Figure 6a). A distinctive feature of cell morphology could be also observed after day 7: only cells in GH-GlcN16 preserved the typical rounded appearance of chondrocytes while cells in GH and GH-GlcN9 appeared more flattened and spindle-like than typical rounded chondrocytes found at day 0 [50]. That chondrocytes in GH-GlcN16 had an almost round shape indicates that cells did not spread well after being cultured for 21 days. In contrast, cells elongated in GH after culturing for 7 days. Chondrocytes cultured in GH-GlcN9 showed a round shape morphology at day 7 but populations of round and spread cells emerged at day 21 of culture.

**Figure 8:**
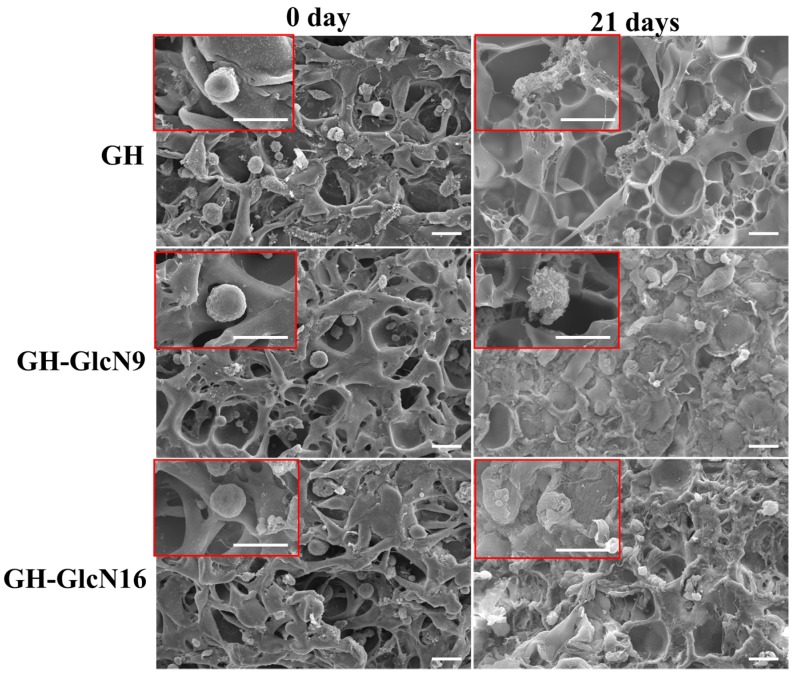
Scanning electron microscopy (SEM) observations of chondrocytes after culturing in GH, GH-GlcN9 and GH-GlcN16 cryogels for 0 and 21 days. The SEM images are shown at 500× and 2500× (inserts) magnifications. Bar = 20 μm.

Cells seeded in cryogels were imaged with SEM at day 0 and 21 (Figure 8). Based on SEM images, we found that cells seeded in all cryogels at day 0 presented the typical round morphology of chondrocytes. After 21 days of cell culture, the ECM secreted by chondrocytes could be identified. In GH scaffolds, only a limited amount of ECM could be identified. However, in GH-GlcN9 and GH-GlcN16 cryogels, abundant matrix deposition was observed to cover the pores of cryogels. In addition, by observing the cell morphology at day 21, we found that cells in GH showed flattened, amoeboid-like shape, indicating that cells were de-differentiated. By contrast, cells in cryogel scaffolds containing GlcN better maintained the polygonal or rounded morphology. Cell shape may play an important role in phenotypic expression in chondrocytes. Cells showing a rounded morphology displayed features of the chondrogenic phenotype: proliferated slowly, incorporated low levels of thymidine into DNA, and incorporated large amounts of SO_4_ into GAGs. In contrast, cells exhibiting flattened morphology were fibroblast-like: faster growth, greater incorporation of thymidine, and less incorporation of SO_4_ [51]. Therefore, incorporation of GlcN in the cryogel facilitated maintaining the normal morphology of chondrocytes and ECM secretion from chondrocytes.

#### 2.4.4. Gene Expression

**Figure 9:**
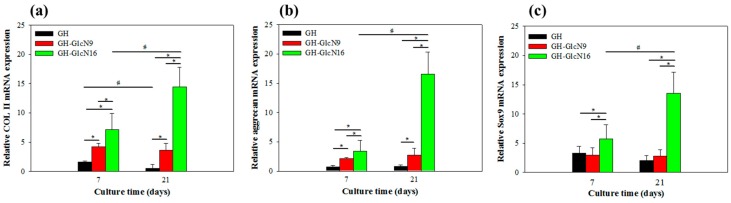
Gene expression of (**a**) type II collagen (COL II), (**b**) aggrecan and (**c**) Sox9 in chondrocytes cultured in GH, GH-GlcN9 and GH-GlcN16 cryogels after 7 and 21 days. * *p* < 0.05 compared between different cryogels; ^#^ *p* < 0.05 compared between different time points.

The chondrogenic gene expression profiles of COL II, aggrecan, and Sox9 are reported in Figure 9. These genes were selected because they are involved in the positive regulation of gene expression in chondrocytes [52,53]. In general, an upregulation was observed for all the genes with increased GlcN content in the cryogel. COL II and Sox 9 gene expression was upregulated for GH-GlcN9 compared with GH. Indeed, GH-GlcN16 is the scaffold containing the most GlcN, and it is also the scaffold for which maximal mRNA level was observed for COL II, aggrecan and Sox9. Comparing results obtained from the two time points, the aggrecan and Sox9 genes showed a constant expression level in GH and GH-GlcN9 from day 7 to day 21. Down-regulation of COL II was observed in GH compared to GH-GlcN9, which could be compared with COL II assays from Figure 6c. In contrast, GH-GlcN16 was the only cryogel presenting enhanced COL II, aggrecan and Sox9 gene expression over time. The reason for this result is again related to the sustained GlcN release from GH-GlcN16 up to 21 days in contrast to only up to 7 days for GH-GlcN9 (Figure 3). With continued stimulation of chondrocytes, enhanced expression level of cartilage-specific genes could be achieved. GlcN has been previously shown to be able to stimulate COL II and aggrecan gene expression of human chondrocytes [54].

Overall, GH-GlcN16 cryogels showed the best performance in terms of maintaining chondrogenic phenotype. This effect could be due to physical cues provided by the unique mechanical properties of GH-GlcN16 when GlcN is introduced (as discussed in Section 3.3), or biological cues offered by GlcN within the cryogel. It is difficult to differentiate between those effects but facile combination of both physical and biological cues with GlcN warrants further in vivo studies using chondrocytes/GH-GlcN16 constructs to repair full-thickness articular cartilage defects in rabbits.

### 2.5. In Vivo Animal Studies

**Figure 10:**
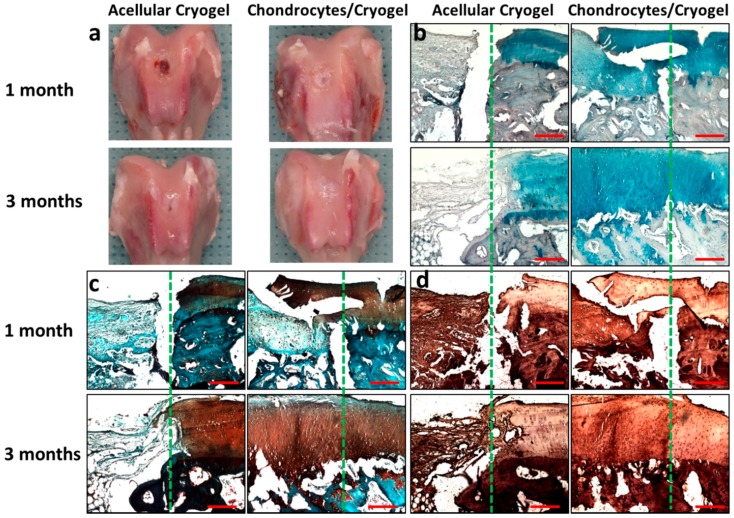
(**a**) Gross observation; (**b**) Alcian blue; (**c**) Safranin O and (**d**) type II collagen immunohistochemical staining of the explanted samples 1 and 3 months post-implantation. The articular cartilage defects of rabbits were repaired with GH-GlcN16 cryogel (acellular cryogel) or chondrocytes-seeded GH-GlcN16 cryogel (chondrocytes/cryogel). The defect creation boundary is shown as the dotted line in each panel with native cartilage to the right. Bar = 200 μm.

We did not observe infection, animal disability or death throughout the in vivo animal experiment. As shown from gross observation in Figure 10a, semi-transparent tissue that was different from native cartilage filled the defect for the acellular cryogel group 1 month post-implantation. The formation of new tissue was scarce and the margin of the defect was visible. After 3 months, cartilage-like tissue of similar colour to the surrounding native cartilage filled the defect, albeit with a visible circular junction between the surrounding and the new tissue. The newly formed tissue also showed rough surface and slight depression at the center. For the chondrocytes/cryogel group, newly formed tissue partially filled the knee joint defect with defined margins and visible edges 1 month post-implantation. After 3 months, the defects were almost completely covered with semi-transparent tissue that was similar to adjacent native cartilage. The distinction between the native cartilage and the neocartilage was difficult and the defect margin disappeared (Figure 10). The quality of newly formed tissue at the site of the defect was semi-quantitatively assessed using the macroscopic ICRS scoring tool 1 and 3 months post-implantation. There is a statistically significant difference between the acellular cryogel and the chondrocytes/cryogel group at both time points (Table 3).

The histological examination with Alcian blue and Safranin O staining are shown in Figure 10b,c. The proteoglycan content in cartilage could be revealed from the intensity of Safranin O staining while acidic polysaccharides such as GAGs in cartilage could be stained by Alcian blue. A thin layer of neocartilage formation could be observed over the residual cryogel in the acellular cryogel group at 1-month. After 3 months, more cartilage formation could be observed from Alcian blue and Safranin O staining; however, the density of lacuna was lower compared with surrounding native cartilage. The integration of native cartilage with newly formed tissue was visible but the defect surface was slightly concave (Figure 10). Neocartilage could be identified from Safranin O and Alcian blue staining to partially fill the defect for the chondrocytes/cryogel group 1 month post-implantation, although the staining intensity was light. The integration between neocartilage and adjacent native cartilage is not complete with gaps easily identified. After 3 months, the neocartilage became much thicker although it is still thinner than surrounding native cartilage. The formation of neocartilage with a typical lacunar structure was evident and the surface was smooth (Figure 10). The integration of new and native cartilage was good with no obvious gap between the neocartilage and surrounding cartilage. Subchondral bone at the defect was also restored. A possible cause may be the fusion of the preformed chondrocytes and surrounding host cartilage [55]. The histological examination results were further subjected to the semi-quantitative ICRS I scoring analysis, which is composed of six sub-items. As shown in Table 3, there are significant differences between the acellular cryogel and the chondrocytes/cryogel group 1 and 3 months post-implantation. 

Figure 10d shows the immunohistochemical staining of COL II for confirmation of the chondrogenic phenotype of implanted chondrocytes. For the acellular group, few areas that stained positive COL II could be identified in the defect area 1 month post-implantation. Much higher staining intensity was found for the chondrocytes/cryogel group at the same time point, although the staining intensity was still less than native cartilage. After 3 months, the acellular group still showed low COL II staining. However, the COL II staining intensity of the implanted chondrocytes/cryogel was similar to that of surrounding native cartilage, indicating that chondrocytes in the cell/scaffold construct continued to preserve the chondrogenic phenotype for neocartilage formation [56]. Taken together, the histology results indicate that chondrocytes in GH-GlcN16 cryogel displayed hyaline-like cartilage regeneration within 3 months with mature chondrocytes showing robust proteoglycan and GAGs contents, and abundant positive COL II staining in the reparative site, which is similar to the adjacent native cartilage.

## 3. Experimental Section

### 3.1. Materials

Sodium hyaluronic acid (HA, average molecular weight = 1.3 MDa) was purchased from Bloomage Freda Biopharm Co., Ltd. (Jinan, China). Gelatin (type A from porcine skin, 300 bloom, average molecular weight 60 kDa), d-(+)-glucosamine hydrochloride, 2-(*N*-morpholino)ethanesulfonic acid (MES), hyaluronidase (Type I-S from bovine testes), antibiotics and trypsin-EDTA were all purchased from Sigma-Aldrich (St. Louis, MO, USA). The compound 1-ethyl-3-(3-dimethylamino-propyl)carbodiimide (EDC) was purchased from Acros (Geel, Belgium). Collagenase (Type I) was purchased from Thermo Fisher Scientific (Waltham, MA, USA). Dulbecco’s Modified Eagle’s Medium/Nutrient Mixture F-12 (DMEM/F-12, Sigma-Aldrich) and fetal bovine serum (FBS, HyClone, GE Healthcare Life Sciences, Logan, UT, USA) were used for cell culture.

### 3.2. Preparation of GH, GH-GlcN9, and GH-GlcN16 Cryogels

HA was dissolved in a 0.1 M MES buffer (pH = 6) at an initial concentration of 0.5% (*w*/*v*). After the HA was completely dissolved, the cross-linking agent EDC was immediately added to reach a final concentration of 4% (*w*/*v*). This solution was mixed at 37 °C for 30 min (solution A). Gelatin at an initial concentration of 10% (*w*/*v*) and GlcN at an initial concentration of 1% or 2% (*w*/*v*) were prepared in 0.1 M MES buffer (pH = 6). The solution was heated at 70 °C until the solute was completely dissolved (solution B). In a 70 °C water bath, solutions A and B were mixed at an equal volume ratio and transferred to a 5-mL plastic syringe with 10 mm internal diameter. The syringe was then placed in 95% alcohol at −16 °C and allowed to react for 16 h. The cryogel scaffold formed within the syringe mold was removed and cut with a sharp blade into disk-shaped pieces (10 mm diameter × 2 mm thickness) and washed with phosphate buffered saline (PBS) for 2 h to remove residual EDC and reaction intermediates, followed by washing with deionized distilled water at 70 °C for 2 h to remove unreacted polymers. Three kinds of cryogels with different compositions (in weight percentage) were prepared: 95% gelatin/5% HA (GH), 87% gelatin/4% HA/9% glucosamine (GH-GlcN9), and 80% gelatin/4% HA/16% glucosamine (GH-GlcN16).

### 3.3. Characterization of Cryogels

Using ethanol as wetting agent, the pore size was determined by capillary flow porometry (PMI CFP-1100-AI, Porous Materials Inc., Ithaca, NY, USA). The ethanol displacement method was used to determine the porosity [57]. The mass of a dried cryogel scaffold over its volume was calculated in order to determine the density. A scanning electron microscope (SEM, Philips XL-30, FEI, Hillsboro, OR, USA) was used to observe the microstructure of the scaffold after gold sputter coating the sample for 60 s.

To determine the swelling in water, the cryogel sample was dried at 60 °C for 24 h in an oven and weighed to obtain the dry weight (*W*_d_). The dried sample was then immersed in deionized water at room temperature for at least 24 h until no measurable mass increase to obtain the equilibrium weight (*W*_eq_) of the swollen sample. The equilibrium swelling ratio was calculated by using Equation (1).

(1) $$\mathrm{Equilibrium}\;\mathrm{swelling}\;\mathrm{ratio}=\left(W_{\mathrm{eq}}-W_{d}\right)/W_{d}$$

The degradation of cryogels was studied in 1% hyaluronidase or 0.1% collagenase solutions prepared in PBS and filtered through 0.22 μm filters. Dried cryogel samples with pre-determined weights (*W*_1_) were UV sterilized and placed in a 24-well plate. Two milliliter enzyme solution was added to each well and incubated in a CO_2_ incubator at 37 °C. The cryogel sample was retrieved at different time points, rinsed with distilled water, and dried in an oven at 70 °C to a constant weight (*W*_2_). The degree of degradation (%) was calculated by using Equation (2).

(2) $$\mathrm{Degree}\;\mathrm{of}\;\mathrm{degradation}=\left(W_{1}-W_{2}\right)/\left(W_{1}\times 100\right)$$

For release of GlcN, a pre-weighed GH-ClcN9 or GH-GlcN16 cryogel sample (10 mm diameter × 2 mm thickness) was UV sterilized and placed in a microcentrifuge tube. One milliliter of spent culture medium from chondrocyte culture at day 3 was added. The tube was placed in a CO_2_ incubator at 37 °C and 200 µL of the medium were removed at intervals and replenished with the same spent medium. The concentration of GlcN was determined using the Elson–Morgan method with an ELISA reader at 530 nm [58] and the cumulative amount of GlcN release was calculated at different time points.

Unconfined compression tests were performed to investigate the mechanical properties of cryogel scaffolds using ElectroForce 5200 BioDynamic Test Instrument from Bose (Eden Prairie, MN, USA). Wet samples were used after soaking cryogels in PBS for 24 h prior to testing. A 250 N compression load was applied at a crosshead speed of 0.02 mm/s. A stress (σ)-strain (ε) curve was recorded with an uniaxial stress. The ultimate stress and strain values were defined as the point where failure of the cryogel occurred. This stress-strain data up to failure was fitted by a non-linear equation (Equation (3)), using fitted parameters A and B [59].

(3) $$\sigma =A\times e^{\left(B\times \varepsilon -1\right)}$$

(4) $$\mathrm{Percent}\;\mathrm{relaxation}=(\sigma_{t}/\sigma_{0})\times 100$$

(5) $$\tan\delta =E^{\prime }/E^{\prime \prime }$$

The elastic modulus at 10% and 30% strain was obtained from the non-linear elastic model from the tangent slope of the stress-strain curve. The area under the stress-strain curve was used to determine the toughness (compressive strain energy to failure), which is defined as the energy necessary to deform a specimen to failure. To evaluate the stress-relaxation behavior, the sample was also compressed to 30% strain in 1 s followed by 600 s relaxation. The remaining stress was plotted as a function of time to get the percent relaxation as specified in Equation (4) where *σ_t_* is stress at time *t* and *σ*_o_ is the initial stress [60]. By loading the sample to 30% strain with 1600 cycles at a frequency of 1 Hz, the cyclic compression test was performed. The stress-strain relation depicted the energy absorption in the cryogel. Dissipation of energy or energy absorbed due to the viscous properties of the cryogel was indicated by a hysteresis loop, bounded by the loading and unloading curves. The area bounded within the hysteresis loop (kJ/m^3^) represents the dissipation energy loss. The energy dissipation percentage (%) was measured by dividing the dissipative energy with the area bounded between the loading curve and the horizontal axis (compression energy), which indicates the total energy applied to the sample during compression. The sample was dynamically tested with sinusoidal compressions from 1 to 6 Hz at 30% compressions for the dynamic compression testing. The dynamic mechanical analysis software measured the complex modulus as the ratio of the stress to the strain, which can be divided into the energy stored per cycle (storage modulus, *E*′) and the energy lost per cycle (loss modulus, *E*′′). The loss tangent (viscous damping) is calculated according to Equation (5).

### 3.4. In Vitro Cell Culture

Knee articular cartilage of rabbits was used to harvest chondrocytes and approved by the Institutional Animal Care and Use Committee of Chang Gung University (IACUC Approval No.: CGU13-035) [61]. Cells at passage 2 were used for cell culture. Disk-shaped cryogel scaffolds (10 mm diameter × 2 mm thickness) were sterilized in a 24-well cell culture plate with 75% ethanol for 24 h followed by UV light exposure overnight in a laminar flow hood. After rinsing in 1 mL PBS for three times, the scaffold was immersed in 1 mL DMEM/F12 for 30 min. Excess medium on the surface of the scaffold was then removed as the scaffold was transferred to a new well in a 24-well cell culture plate for cell seeding. An aliquot of 20 μL cell suspension (2 × 10^6^ cells/mL) was seeded directly onto the top surface of the wet cryogel disk. The cell-seeded cryogel was incubated at 37 °C in a CO_2_ incubator for 2 h to allow cell attachment and turned upside down for cell seeding to the other surface as before. After incubating for 2 h in a CO_2_ incubator, the cell-seeded scaffold was transferred to a new well in a 24-well cell culture plate and 2 mL of DMEM/F12 containing 10% FBS and 1.0% streptomycin–penicillin solution was added to each well and cultured at 37 °C in humidified 5% CO_2_ for 21 days with medium change every 3 days. 

The cell/scaffold constructs were harvested at predetermined times and digested in 1 mL papain solution (55 mM sodium citrate, 150 mM sodium chloride, 5 mM cysteine HCl, 5 mM EDTA and 0.2 mg/mL papain) at 60 °C for 24 h to determine the DNA, GAGs and COL II contents. Hoechst 33258 was used to determine the DNA content [62], while 1,9-dimethylmethylene blue was used to determine the GAGs content with chondroitin-6-sulfate as the standard [63]. Quantitatively determination of COL II content was carried out by sandwich ELISA using rabbit anti-COL II polyclonal antibody (Bioss bs-0709R, Woburn, MA, USA) and HRP-conjugated rabbit anti-COL II polyclonal antibody (Bioss bs-4851R-HRP).

For SEM observation, the chondrocyte/cryogel constructs cultured for 21 days were fixed with 2.5% glutaraldehyde for 24 h at room temperature. After thorough washing with 0.1 M PBS (pH = 7.4), the samples were dehydrated in ethanol in a sequential manner (50%, 70%, 80%, 90%, and 95%) for 15 min each, immersed in 99.5% ethanol for 20 min, dried in a critical point dryer (LEICA EM CPD300, Wetzlar, Germany), and observed by SEM (JEOL ISM-5410, Tokyo, Japan) after gold coating.

The qualitative evaluation on the cell viability of chondrocytes was assessed using the Live/Dead viability/cytotoxicity kit (Molecular Probes, Eugene, OR, USA). After culturing for 7 and 21 days, the medium was removed and samples were washed three times with PBS. The Live/Dead staining solution was prepared with 3 μL of 4 mM calcein AM (excitation 494 nm and emission 517 nm) and 5 μL of 2 mM ethidium homodimer-1 (EthD-1) (excitation 528 nm and emission 617 nm) in 10 mL PBS to detect live and dead cells separately. All samples were incubated in 300 μL of staining solution for 10 min and imaged under a Zeiss LSM 510 Meta Confocal Laser Scanning Microscope (Jena, Germany).

The expression of cartilage-specific marker genes was examined using standard protocols of RNA isolation and cDNA synthesis [6]. Quantitative real-time polymerase chain reaction (qRT-PCR) measurements were performed using a SYBR Green RT-PCR kit (SYBR Green I supermix, Bio-Rad) in a MiniOpticon™ real-time PCR detection system (Bio-Rad CFD-3120). Type II collagen (COL II), aggrecan and Sox 9 cartilage-specific genes were selected for analysis and quantified using the delta-delta *C*_t_ relative quantification method. Glyceraldehyde-3-phosphate dehydrogenase (GAPDH) acted as a housekeeping control. The primers (Watson Biotechnology Co., Ltd., Taipei, Taiwan) used were COL II (forward: 5′-GCCACCGTGCCCAAGAAGAACT-3′; reverse: 5′-ACAGCAGGCGCAGGAAGGTCAT-3′), aggrecan (forward: 5′-CCTACCAGGACAAGGTCTCG -3′; reverse: 5′-ACACCTTTCACCACGACCTC-3′), Sox9 (forward: 5′-GGAAGCTCTGGAGAC TGCTG-3’; reverse: 5’-CGTTCTTCACCGACTTCCTC-3′) and GAPDH (forward: 5′-ATCACT GCCACCCAGAAGAC-3′; reverse: 5′-GTGAGTTTCCCGTTCAGCTC-3′).

### 3.5. In Vivo Animal Study

The Institutional Animal Care and Use Committee of Chang Gung University approved the animal protocols (IACUC Approval No.: CGU13-035). They are in accordance with the standards of the Association for Assessment and Accreditation of Laboratory Animal Care. Ketamine (20 mg/kg) was administered through intramuscular injection for anesthesia before animal surgery. The knee area of the rabbit’s hind limb was shaved and prepared. Four percent isoflurane was used for induction while maintenance of general anesthesia was obtained through the administration of 2% isoflurane with an O_2_ mask at 2.5 L/min. The rabbit was placed in supine position and the surgical field was sterilized using iodine solutions and the non-sterile area was covered with a sterile drape. All surgical instruments were sterilized and kept sterile throughout the procedure. The knee joint was opened through a 5.0-cm medial parapatellar incision approach. An electric drill equipped with a 4-mm diameter drill bit was used to create full-thickness defects (4 mm diameter × 2 mm thickness) through the articular cartilage and subchondral bone of the central patellar groove. Twelve rabbits were randomly divided into two groups. Defects in the acellular group were implanted with disk-shaped GH-GlcN16 (4 mm diameter × 2 mm thickness) while defects in the chondrocytes/cryogel group were implanted with chondrocytes/GH-GlcN16 constructs of the same size, which were pre-seeded with 1 × 10^5^ chondrocytes and cultured in DMEM/F12 containing 10% FBS and 1.0% streptomycin–penicillin solution for 14 days. 4-0 Ethicon sutures were used to close the skin after operation and gentamicin prophylactic antibiotics were administered. The wounds were sterilized and further covered with gentamicin ointments to prevent infection. There were no movement restrictions for any of the 12 rabbits post-operation. Three animals from each group were euthanized with lethal doses of pentobarbital (0.5 g/kg) at 1 and 3 months postsurgery. The whole joint surfaces including the implants were harvested for examination and photography. For gross evaluation, the surface of the osteochondral defect areas were blindly evaluated by two surgeons using the International Cartilage Repair Society (ICRS) score [64]. The scoring system includes three criteria with the score in the range of 0 to 4 in each category. After gross evaluation, the samples were fixed in 10% formaldehyde, dehydrated in alcohol and embedded in paraffin, and sections were stained by Alcian blue, Safranin O, and immunohistochemical staining of COL II. The histology sections were blindly scored by two pathologists using the ICRS I score [65]. The scoring system includes six criteria with the score ranging from 0 to 3 in each category.

### 3.6. Statistical Analysis

All data are reported as mean ± standard deviation (SD). One-way ANOVA LSD test was used among multiple groups, while Tukey’s post hoc test was used to determine the difference between any two groups using the SPSS software (SPSS Inc., Chicago, IL, USA). A *p* value <0.05 was considered statistically significant.

## 4. Conclusions

Macroporous GH and GH-GlcN cryogels with 9 and 16 wt % GlcN were prepared at −16 °C using EDC as a crosslinking agent. Physico-chemical properties evaluation revealed similar pore size, but higher porosity, degradation, and swelling ratio after incorporation of GlcN. GlcN could modulate the mechanical properties of cryogels. The Young’s modulus, storage modulus, ultimate compressive stress, energy dissipation level, and rate of stress relaxation decreased while the elasticity increased by increasing the GlcN content in the cryogel, which is believed to be related to the low crosslinking density when gelatin is substituted with GlcN. Many of the unique properties of GH-GlcN16 are deemed beneficial for maintaining chondrogenic phenotype. The release of GlcN from the scaffolds in the culture medium of chondrocytes could be sustained for 21 days for GH-GlcN16 in contrast to only 7 days for GH-GlcN9. In vitro cell culture experiments indicated that GH-GlcN16 showed the best effects on maintaining chondrogenic phenotype with reduced cell proliferation but with the highest levels of GAGs and COL II production. Gene expression analysis also showed time-dependent up-regulation of cartilage-specific marker genes (COL II, aggrecan and Sox9) for chondrocytes in GH-GlcN16. In the rabbit full-thickness articular cartilage defect model, chondrocytes/GH-GlcN16 constructs could regenerate neocartilage with positive staining for GAGs and COL II compared with acellular GH-GlcN16. We concluded that one function of GlcN is to modulate scaffold properties that are beneficial for chondrocytes. Combining such physical cues provided by cryogel scaffolds and the other function of GlcN, which serves as biological cues for maintaining chondrogenic phenotype in vitro, GH-GlcN16 cryogel was demonstrated to be a suitable scaffold for cartilage tissue engineering in vivo.

## Figures and Tables

**Table 1:** Properties of cryogel scaffolds. Values are mean ± standard deviation (SD) of six independent measurements.

	GH	GH-GlcN9	GH-GlcN16
Pore size (μm)	103.5 ± 9.4	105.6 ± 11.5	108.3 ± 13.3
Porosity (%)	80.9 ± 0.7	83.8 ± 1.8 *	91.1 ± 1.5 *^,#^
Density (mg/cm^3^)	134.9 ± 1.2	131.5 ± 1.0 *	127.6 ± 1.3 *^,#^
Swelling ratio	11.4 ± 0.9	19.2 ± 0.6 *	31.4 ± 1.7 *^,#^

GH: gelatin/hyaluronic acid cryogel; GH-GlcN9: gelatin/hyaluronic acid/glucosamine cryogel with 9% glucosamine; GH-GlcN16: gelatin/hyaluronic acid/glucosamine cryogel with 16% glucosamine. * *p* < 0.05 compared with GH; ^#^ *p* < 0.05 compared with GH-GlcN9.

**Table 2:** Mechanical properties of GH, GH-GlcN9 and GH-GlcN16 cryogels. Values are mean ± SD of five independent measurements.

	GH	GH-GlcN9	GH-GlcN16
Compressive elastic modulus, *ε* = 0.1 (kPa)	45.1 ± 5.3	30.3 ± 4.1 *	24.9 ± 3.6 *
Compressive elastic modulus, *ε* = 0.3 (kPa)	255.6 ± 77.9	156 ± 25.3	132.3 ± 19.8 *
Compressive strain to failure (%)	45.7 ± 1.5	49 ± 1.0	47.7 ± 3.1
Compressive stress to failure (kPa)	118.6 ± 12.1	90.6 ± 8.9 *	53.3 ± 5.0 *^,#^
Toughness (kJ/m^3^)	12.7 ± 1.3	10.8 ± 0.8	8.2 ± 2.1 *
Compression energy (kJ/m^3^)	2.5 ± 0.1	1.9 ± 0.3 *	1.3 ± 0.1 *^,#^
Relaxation energy (kJ/m^3^)	0.8 ± 0.1	0.7 ± 0.2	0.5 ± 0.1 *
Dissipation energy (kJ/m^3^)	1.7 ± 0.1	1.2 ± 0.1 *	0.8 ± 0.1 *^,#^
Percentage of energy dissipation (%)	68.2 ± 4.4	61.3 ± 1.4 *	59.7 ± 1.6 *

* *p* < 0.05 compared with GH cryogel; ^#^ *p* < 0.05 compared with GH-GlcN9 cryogel.

**Table 3:** Semi-quantitative analysis of articular cartilage defect repair 1 and 3 months post-implantation by ICRS scores. Values are mean ± SD from six analyses.

	Acellular Cryogel		Chondrocytes/Cryogel	
	Gross View	Histology	Gross View	Histology
1 month	2.83 ± 0.98	2.67 ± 0.82	5.33 ± 0.82 *	7.17 ± 1.17 *
3 months	4.83 ± 0.41 *	5.50 ± 0.84 *	8.33 ± 1.03 ^#^	14.83 ± 2.40 ^#^

Acellular cryogel: gelatin/hyaluronic acid/glucosamine cryogel with 16% glucosamine (GH-GlcN16) cryogel. Chondrocytes/cryogel: chondrocytes-seeded GH-GlcN16 cryogel. * *p* < 0.05 compared with acellular cryogel at 1-month; ^#^ *p* < 0.05 compared with acellular cryogel at 3-months. The maximum scoring scales are 12 and 18 for gross view and histology, respectively. ICRS: International Cartilage Repair Society.
